# Different definitions of esophagus influence esophageal toxicity prediction for esophageal cancer patients administered simultaneous integrated boost versus standard-dose radiation therapy

**DOI:** 10.1038/s41598-017-00168-x

**Published:** 2017-03-09

**Authors:** Bao-tian Huang, Rui-hong Huang, Wu-zhe Zhang, Wen Lin, Long-jia Guo, Liang-yu Xu, Pei-xian Lin, Jian-zhou Chen, De-rui Li, Chuang-zhen Chen

**Affiliations:** 1 Department of Radiation Oncology, Cancer Hospital of Shantou University Medical College, Shantou, China; 2 Department of Respiratory Medical Oncology, Cancer Hospital of Shantou University Medical College, Shantou, China; 3 Department of Nosocomial Infection Management, The Second Affiliated Hospital of Shantou University Medical College, Shantou, China; 4 CRUK/MRC Oxford Institute for Radiation Oncology, University of Oxford, Oxford, UK

## Abstract

We aim to evaluate whether different definitions of esophagus (DEs) impact on the esophageal toxicity prediction for esophageal cancer (EC) patients administered intensity-modulated radiation therapy with simultaneous integrated boost (SIB-IMRT) vs. standard-dose IMRT (SD-IMRT). The esophagus for 21 patients diagnosed with primary EC were defined in the following four ways: the whole esophagus, including the tumor (ESO_whole_); ESO_whole_ within the treatment field (ESO_infield_); ESO_infield_, excluding the tumor (ESO_infield-tumor_) and ESO_whole_, excluding the tumor (ESO_whole-tumor_). The difference in the dose variation, acute esophageal toxicity (AET) and late esophageal toxicity (LET) of four DEs were compared. We found that the mean esophageal dose for ESO_whole_, ESO_infield_, ESO_infield-tumor_ and ESO_whole-tumor_ were increased by 7.2 Gy, 10.9 Gy, 4.6 Gy and 2.0 Gy, respectively, in the SIB-IMRT plans. Radiobiological models indicated that a grade ≥ 2 AET was 2.9%, 3.1%, 2.2% and 1.6% higher on average with the Kwint model and 14.6%, 13.2%, 7.2% and 3.4% higher with the Wijsman model for the four DEs. A grade ≥ 3 AET increased by 4.3%, 7.2%, 4.2% and 1.2%, respectively. Additionally, the predicted LET increased by 0.15%, 0.39%, 1.2 × 10^−2^% and 1.5 × 10^−3^%. Our study demonstrates that different DEs influence the esophageal toxicity prediction for EC patients administered SIB-IMRT vs. SD-IMRT treatment.

## Introduction

Recently, an increasing number of clinical studies have demonstrated the feasibility, efficacy and safety of intensity-modulated radiation therapy with simultaneous integrated boost (SIB-IMRT) for the treatment of esophageal cancer (EC), resulting in an improvement in the local-regional control and overall survival while maintaining clinically tolerated toxicities^1–3^.

Although the SIB-IMRT technique has shown encouraging outcomes for EC patients, concerns about the increased risk of esophageal toxicity with the SIB-IMRT technique are increasing. Acute esophageal toxicity (AET) and late esophageal toxicity (LET) are two common dose-limiting toxicities that will hinder the use of dose escalation in EC patients administered concurrent chemoradiotherapy (CCRT)^2, 4, 5^. Unfortunately, few clinical investigations have been conducted to explore the potential risk of esophageal toxicity using SIB-IMRT vs. standard-dose IMRT (SD-IMRT). Furthermore, most of the studies on the risks of esophageal toxicity induced by IMRT and concurrent chemotherapy strategies were primarily conducted on lung cancer patients^4, 6, 7^. In contrast, EC patients are particularly different from lung cases because the tumor is within the esophagus, resulting in the heterogeneity of the esophagus definition when implementing the radiation therapy treatment. Therefore, whether different definitions of esophagus (DEs) influence the evaluation of the esophageal toxicity in response to SIB-IMRT vs. SD-IMRT should be further investigated.

In this study, we aim to evaluate the impact of DEs on the dosimetric changes and esophageal toxicity prediction between the SIB-IMRT and SD-IMRT dosing strategies in EC patients using predictive models derived from clinical data.

## Materials and Methods

### Ethics statement

All experimental protocols were approved by the Clinical Research Ethics Review Committee of Cancer Hospital of Shantou University Medical College. All methods in this study were conducted in accordance with the relevant guidelines and regulations. Considering that this is not a treatment-based study, our institutional review board waived the need for obtaining written informed consent from the participants.

### Patient selection

CT simulating data sets of 21 upper thoracic esophageal cancer patients enrolled in a phase II clinical trial (Clinicaltrial.gov number, NCT01670409, and Chinese Clinical Research Registry number, ChiCTR-ONC-12002356) were used in this study.

### Immobilization and simulation

The patients were immobilized in supine position with the head and shoulders wrapped in a thermoplastic mask (Guangzhou Klarity Medical & Equipment Co., Ltd, Guangzhou, China). Contrast enhanced computer tomography (CT) scans of 0.5-mm slice thickness from the neck to the upper abdomen were obtained using a 16-slice CT scanner (Philips Brilliance Big Bore Oncology Configuration, Cleveland, OH) under free breathing. The CT images were subsequently delivered to the Eclipse Treatment Planning System (Version 10.0, Varian Medical Systems, Palo Alto, CA) by DICOM (Digital Imaging and Communications in Medicine) 3.0 interface for target volume contouring, organs at risk (OARs) contouring and treatment planning.

### Target volume and OARs delineation

We previously introduced the methods of target and OARs delineation for EC patients^8^. Briefly, the gross tumor volume (GTV) included the primary tumor (GTV_P_) and positive regional lymph nodes (GTV_LN_). The delineation of GTV was determined using CT images, endoscopic reports or barium swallow fluoroscopy. GTV_LN_ included mediastinal or supraclavicular lymph nodes with the shortest axis ≥1 cm. Clinical target volume (CTV) was delineated with a 2-cm margin in the longitudinal direction and a 0.5–1.0 cm margin in the radial direction with respect to the GTV_P_ and a 0.5-cm uniform margin from GTV_LN_. Paraesophageal or tracheoesophageal groove lymph nodes that did not meet the criteria of positive lymph nodes, but with their shortest axis ≥0.5 cm were also encompassed in CTV. To generate two planning target volume (PTV), PTV64.8 and PTV50.4, an isotropic 0.5-cm margin was expanded from GTV and CTV, respectively. OARs, including spinal cord and lung were generated according to the reference^9^. Briefly, lung contouring was limited to the air-inflated lung parenchyma without inclusion of the fluid and atelectasis visible on CT images. The proximal bronchial tree should also be excluded. Contouring of the spinal cord should start at the same cranial level as the esophagus to the bottom of L2, or the level at which the cord ended. The planning OAR volume (PRV) for the spinal cord was generated from the spinal cord expanding a 0.5-cm margin. Four types of esophagus delineation were generated to identify their dose-response differences. ESO_whole_ presented the whole esophagus from the level of cricoid cartilage on every CT image to the gastroesophageal junction, including the tumor^6, 7, 10, 11^; ESO_infield_ was the portion of ESO_whole_ within the treatment field, where the treatment field was defined as the upper and lower edges of the largest fields^10^; ESO_infield-tumor_ was the portion of ESO_infield_, excluding the tumor; and ESO_whole-tumor_ was the portion of ESO_whole_, also excluding the tumor.

### Planning objectives

The following dose constraints for OARs were used: spinal cord, D_max_ (maximum dose) <45 Gy; PRV for spinal cord, V_50_ ≤ 1 cc; lung, V_5_ < 60%, V_10_ < 50%, V_20_ < 30% and mean lung dose (MLD) < 15 Gy, where V_x_ is percentage of the target volume receiving ≥ x Gy dose. The dose was normalized to ensure that 95% of the PTV received 100% of the prescription.

### Treatment planning

The prescription for the SIB-IMRT plan was set at 64.8 Gy in 28 fractions for PTV64.8 (delivered in 2.31 Gy/fraction) and 50.4 Gy in 28 fractions for PTV50.4 (delivered in 1.8 Gy/fraction)^12^. The prescription for the SD-IMRT plans was set at 50.4 Gy in 28 fractions (delivered in 1.8 Gy/fraction) for PTV50.4. Treatment plans were generated using five sliding window-based coplanar fields, with beam arrangements of 210°, 300°, 0°, 60° and 150°. All plans were designed using 6 MV photon beam from a TrueBeam linear accelerator (Varian Medical Systems, Palo Alto, CA). Plan optimization was performed using the Dose Volume Optimizer (DVO, version 10.0.28) algorithm, selecting a maximum dose rate of 600 monitor units per minute (MU/min). The dose calculation was performed using the Anisotropic Analytical Algorithm (AAA, version 10.0.28), considering the heterogeneity correction. Several dose-limiting structures were generated to make the dose conformal to the target. We employed the base dose function (BDF) method as reported in our previous study to acquire a more homogeneous dose distribution^13^. Briefly, the fractions of the original plan were modified to half (from 28 to 14 in the study), and subsequently, the half-prescribed plan was copied and reoptimized using the half-prescribed plan as the base dose. After the dose was calculated, the fractions of the plan were doubled to generate the target plan.

### Prediction of esophageal toxicity

We used the Kwint model to predict grade ≥ 2 and grade ≥ 3 AET^7^. The Kwint model derived from 139 patients after CCRT treatment for patients with non-small cell lung cancer (NSCLC) shows a sigmoid-shaped relationship between grade ≥ 2 AET and V_50_. Moreover, the Wijsman model, which is a Lyman-Kutcher-Burman (LKB)-based predicting model generated from 149 advanced stage NSCLC patients undergoing CCRT, was also established for estimating grade ≥ 2 AET^6^. The following parameters were used in the Wijsman model: n = 1.04, m = 0.65 and D_50_ = 32.84 Gy. The Chen model is an a LKB-based predicting model derived from 171 patients NSCLC patients treated with CCRT, and we use it to predict the incidence of LET^4^ using the following parameters: n = 0.03, m = 0.03 and TD_50_ = 76.1 Gy. All physical doses were converted to a biologically equivalent dose in 2 Gy fraction (EQD_2_) dose to calculate the potential risk. For esophageal toxicity prediction, α/β of 10 and 3 Gy were employed to predict AET and LET, respectively. A detailed procedure of this calculation was published in our previous work^14^.

### Statistical analysis

All data in this study were shown as the mean plus standard deviation (mean ± SD). Data analysis was performed using SPSS version 19.0 software (SPSS, Inc., Chicago, IL, USA). The Friedman Test was used to determine the difference in dosimetry and predicted toxicity among four DEs. Comparison of the sub-group data was compared using the Wilcoxon signed-rank test. The results were considered statistically significant at a *p*-value < 0.05.

## Results

### Patient characteristics

From September 2012 to December 2013, 21 upper thoracic esophageal cancer patients were used in this study. The age of the patients ranged from 49 to 73 years old and the other characteristics were listed in Table 1.

**Table 1:** Basic characteristics of 21 patients with EC.

Patient	Gender	Age	Stage*
1	M	53	T3N1M0
2	M	64	T3N1M1
3	M	49	T3N1M0
4	M	64	T3N0M0
5	M	55	T3N1M0
6	M	73	T2N0M0
7	M	61	T3N1M0
8	M	59	T2N1M0
9	M	61	T4N0M1
10	M	59	T3N1M0
11	M	56	T4N1M0
12	F	53	T2N0M0
13	M	60	T4N1M0
14	M	64	T3N1M0
15	M	72	T3N0M0
16	M	66	T4N0M0
17	M	59	T4N0M0
18	M	67	T3N0M0
19	F	67	T2N1M1
20	F	65	T3N0M0
21	M	69	T2N1M0

*Abbreviations:* M = Male; F = Female.

*Note:* *According to American Joint Committee on Cancer (AJCC), 6^th^ edition.

### DEs influence dose changes for EC patients administered SIB-IMRT vs. SD-IMRT

**Figure 1:**
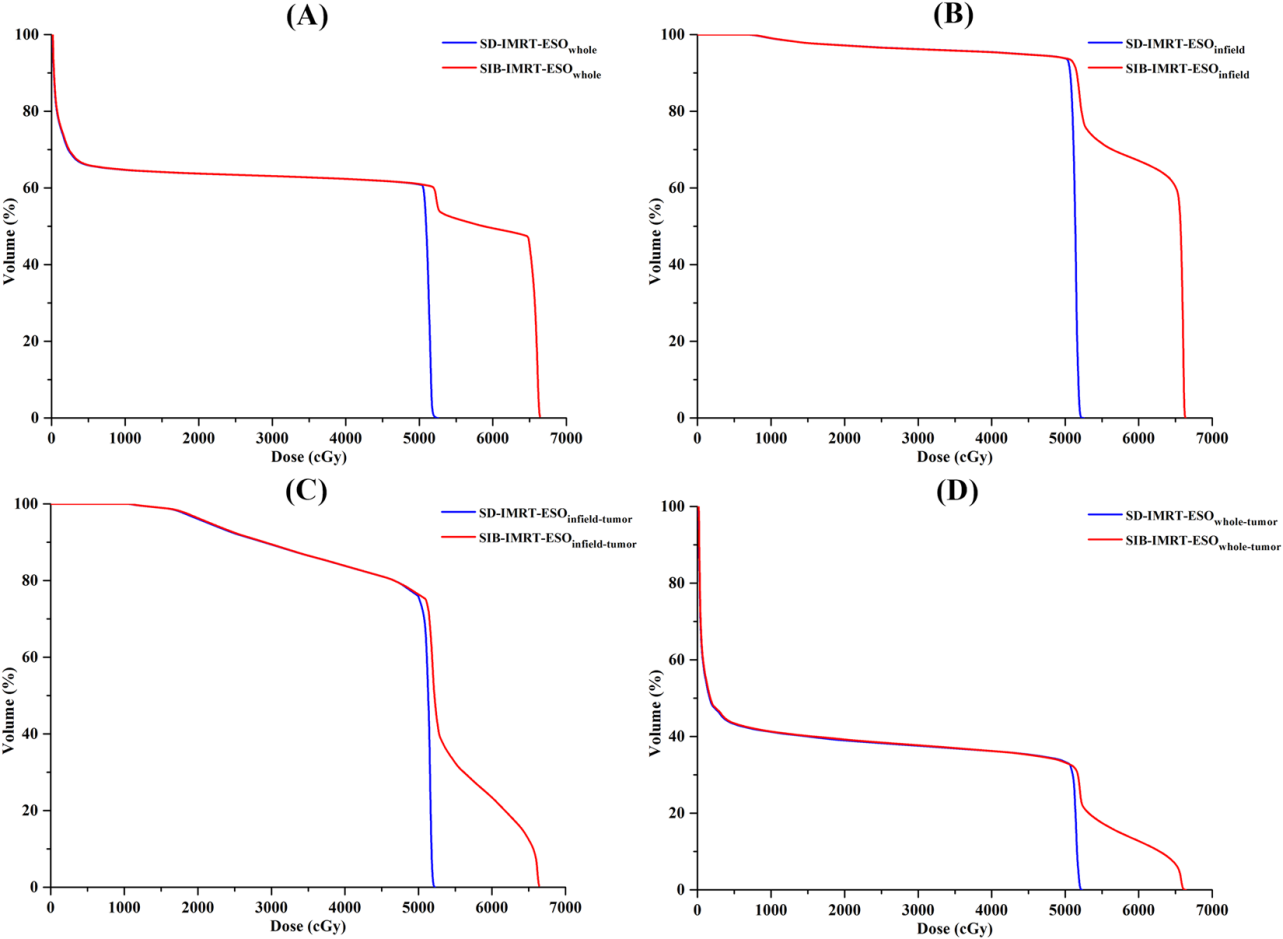
DVH for different DEs. (**A**) DVH for ESO_whole_, **(B**) DVH for ESO_infield_, (**C**) DVH for ESO_infield-tumor_ and (**D**) DVH for ESO_whole-tumor_. ESO_whole_ = the entire esophagus including the tumor; ESO_infield_ = the portion of ESO_whole_ within the treatment field; ESO_infield-tumor_ = the portion of ESO_infield_, excluding the tumor; ESO_whole-tumor_ = the portion of ESO_whole_, excluding the tumor. SIB-IMRT = intensity-modulated radiation therapy with simultaneous integrated boost; SD-IMRT = standard-dose intensity-modulated radiation therapy.

**Figure 2:**
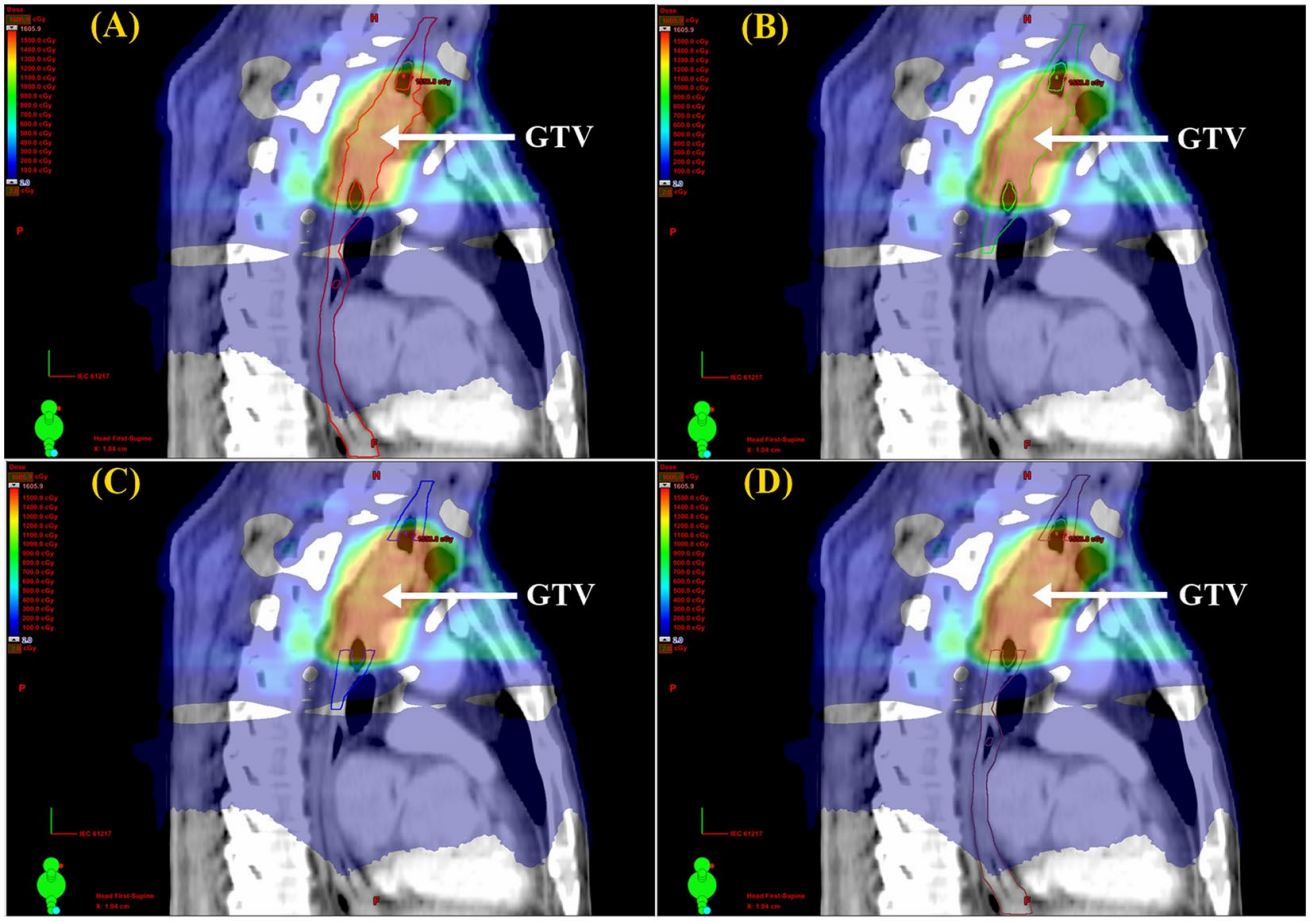
Esophageal dose variation for four DEs. Color wash displayed the dose difference between the SIB-IMRT and SD-IMRT dosing strategies from one representative case. (**A**) Dose for ESO_whole_ (red line), (**B)** dose for ESO_infield_ (green line), (**C**) dose for ESO_infield-tumor_ (blue line), (**D**) dose for ESO_whole-tumor_ (brown line). ESO_whole_ = the entire esophagus, including the tumor; ESO_infield_ = the portion of ESO_whole_ within the treatment field; ESO_infield-tumor_ = the portion of ESO_infield_ excluding the tumor; ESO_whole-tumor_ = the portion of ESO_whole_ excluding the tumor. SIB-IMRT = intensity-modulated radiation therapy with simultaneous integrated boost; SD-IMRT = standard-dose intensity-modulated radiation therapy.

The dose differences of the four DEs were listed in Table 2. Compared with the SD-IMRT plan, different DEs resulted in similar dose increase in the SIB-IMRT plan. The increase of V_30_, V_40_ and V_50_ for the four DEs was comparable (*p* > 0.05). However, the increase of V_60_ and D_mean_ was significantly different (*p* < 0.05). Specifically, V_60_ values for the ESO_whole_, ESO_infield_, ESO_infield-tumor_ and ESO_whole-tumor_ were increased by 29.6 cc, 29.5 cc, 3.4 cc and 3.7 cc, respectively. D_mean_ for ESO_whole_, ESO_infield_, ESO_infield-tumor_ and ESO_whole-tumor_ were increased by 7.2 Gy, 10.9 Gy, 4.6 Gy and 2.0 Gy, respectively. However, D_max_ values for the ESO_whole_, ESO_infield_, ESO_infield-tumor_ and ESO_whole-tumor_ were equally increased by 14.2 Gy. Compared with ESO_whole-tumor_ and ESO_infield-tumor_, the increase of V_60_ and D_mean_ was higher for the ESO_whole_ and ESO_infield_ definitions. The dose volume histogram (DVH) for the four DEs was presented in Fig. 1. The dose variation for the four DEs in the sagittal view from one representative case was illustrated in Fig. 2.

**Table 2:** Esophageal dose changes for four DEs.

Strategy	Parameters	ESO_whole_	ESO_infield_	ESO_infield-tumor_	ESO_whole-tumor_	*p*	*p* ^*1*^	*p* ^*2*^
SIB-IMRT	V_30_ (cc)	38.1 ± 17.8	37.7 ± 17.1	11.7 ± 3.1	12.2 ± 3.4	N/A	N/A	N/A
	V_40_ (cc)	37.6 ± 17.8	37.2 ± 17.0	11.2 ± 3.0	11.6 ± 3.3	N/A	N/A	N/A
	V_50_ (cc)	36.7 ± 17.8	36.3 ± 17.0	10.3 ± 2.7	10.7 ± 3.1	N/A	N/A	N/A
	V_60_ (cc)	29.6 ± 16.7	29.5 ± 16.3	3.4 ± 1.1	3.7 ± 1.4	N/A	N/A	N/A
	D_mean_ (Gy)	40.5 ± 7.6	60.6 ± 2.6	51.8 ± 4.1	22.6 ± 4.9	N/A	N/A	N/A
	D_max_ (Gy)	66.7 ± 0.3	66.7 ± 0.3	66.7 ± 0.3	66.7 ± 0.3	N/A	N/A	N/A
SD-IMRT	V_30_ (cc)	38.1 ± 17.8	37.7 ± 17.1	11.7 ± 3.1	12.1 ± 3.4	N/A	N/A	N/A
	V_40_ (cc)	37.6 ± 17.8	37.2 ± 17.0	11.2 ± 3.0	11.6 ± 3.3	N/A	N/A	N/A
	V_50_ (cc)	36.7 ± 17.7	36.3 ± 17.0	10.2 ± 2.6	10.7 ± 3.0	N/A	N/A	N/A
	V_60_ (cc)	0.0 ± 0.0	0.0 ± 0.0	0.0 ± 0.0	0.0 ± 0.0	N/A	N/A	N/A
	D_mean_ (Gy)	33.3 ± 5.7	49.8 ± 1.7	47.2 ± 3.4	20.6 ± 4.3	N/A	N/A	N/A
	D_max_ (Gy)	52.5 ± 0.4	52.5 ± 0.4	52.5 ± 0.4	52.5 ± 0.4	N/A	N/A	N/A
SIB-SD	V_30_ (cc)	0.0 ± 0.1	0.0 ± 0.1	0.0 ± 0.1	0.0 ± 0.1	0.881	0.655	0.317
	V_40_ (cc)	0.0 ± 0.1	0.0 ± 0.1	0.0 ± 0.1	0.0 ± 0.1	0.674	0.492	0.228
	V_50_ (cc)	0.1 ± 0.2	0.1 ± 0.2	0.1 ± 0.2	0.1 ± 0.2	0.815	0.739	1.000
	V_60_ (cc)	29.6 ± 16.7	29.5 ± 16.3	3.4 ± 1.1	3.7 ± 1.4	0.000	0.000	0.000
	D_mean_ (Gy)	7.2 ± 1.9	10.9 ± 1.3	4.6 ± 1.2	2.0 ± 0.8	0.000	0.000	0.000
	D_max_ (Gy)	14.2 ± 0.3	14.2 ± 0.3	14.2 ± 0.3	14.2 ± 0.3	0.001	0.066	0.066

*Abbreviations:* SIB-IMRT = intensity-modulated radiation therapy with simultaneous integrated boost; SD-IMRT = standard-dose intensity-modulated radiation therapy; SIB-SD = the difference between the SIB-IMRT and SD-IMRT plans; ESO_whole_ = the entire esophagus, including the tumor; ESO_infield_ = the portion of ESO_whole_ within the treatment field; ESO_infield-tumor_ = the portion of ESO_infield_, excluding the tumor; ESO_whole-tumor_ = the portion of ESO_whole_, excluding the tumor. D_mean_ = mean dose; D_max_ = maximum dose; V_x_ = the volume of the organ receiving a dose of x or more. N/A = not available. *p* ^*1*^: ESO_whole_ vs. ESO_whole-tumor_; *p* ^*2*^ ESO_infield_ vs. ESO_infield-tumor_.

### DEs impact on the esophageal toxicity prediction for EC patients administered SIB-IMRT vs. SD-IMRT

The esophageal toxicity prediction for the four DEs was listed in Table 3. Compared with the SD-IMRT plan, the predicted grade ≥ 2 AET values using the Kwint model in the SIB-IMRT group were 2.9%, 3.1%, 2.2% and 1.6% higher on average for ESO_whole_, ESO_infield_, ESO_infield-tumor_ and ESO_whole-tumor_, respectively. Grade ≥ 2 AET predicted using the Wijsman predicting model were 14.6%, 13.2%, 7.2% and 3.4% higher on average. Additonally, the SIB-IMRT plan was also 4.3%, 7.2%, 4.2% and 1.2% higher on average for grade ≥ 3 AET. The predicted LET were increased by 0.15%, 0.39%, 1.2 × 10^−2^% and 1.5 × 10^−3^% for the four DEs. Compared with ESO_whole-tumor_ and ESO_infield-tumor_, the increase was higher with the ESO_whole_ and ESO_infield_ definition.

**Table 3:** Esophageal toxicity prediction for four Des.

Strategy	Parameters	ESO_whole_	ESO_infield_	ESO_infield-tumor_	ESO_whole-tumor_	*p*	*p* ^*1*^	*p* ^*2*^
SIB-IMRT	Kwint (%)^a^	75.1 ± 5.7	87.9 ± 1.5	83.3 ± 3.5	59.2 ± 4.2	N/A	N/A	N/A
	Kwint (%)^b^	37.2 ± 8.7	61.0 ± 3.8	50.0 ± 6.9	19.2 ± 3.2	N/A	N/A	N/A
	Wijsman (%)^c^	63.5 ± 13.6	90.8 ± 2.5	80.7 ± 6.1	30.2 ± 7.7	N/A	N/A	N/A
	Chen (%)^d^	(1.5 ± 1.0) × 10^−1^	(3.9 ± 1.6) × 10^−1^	(1.2 ± 2.8) × 10^−2^	(1.5 ± 5.0) × 10^−3^	N/A	N/A	N/A
SD-IMRT	Kwint (%)^a^	72.2 ± 5.8	84.8 ± 2.1	81.1 ± 3.9	57.7 ± 4.2	N/A	N/A	N/A
	Kwint (%)^b^	32.9 ± 7.7	53.8 ± 4.7	45.8 ± 7.0	18.0 ± 2.9	N/A	N/A	N/A
	Wijsman (%)^c^	48.8 ± 10.7	77.6 ± 2.7	73.5 ± 5.8	26.8 ± 6.6	N/A	N/A	N/A
	Chen (%)^d^	(1.7 ± 1.7) × 10^−23^	(1.4 ± 1.2) × 10^−22^	(8.1 ± 5.3) × 10^−23^	(6.8 ± 6.0) × 10^−25^	N/A	N/A	N/A
SIB-SD	Kwint (%)^a^	2.9 ± 1.6	3.1 ± 1.8	2.2 ± 1.4	1.6 ± 1.0	0.000	0.002	0.048
	Kwint (%)^b^	4.3 ± 2.7	7.2 ± 3.8	4.2 ± 2.8	1.2 ± 0.9	0.000	0.000	0.008
	Wijsman (%)^c^	14.6 ± 3.1	13.2 ± 1.0	7.2 ± 1.2	3.4 ± 1.2	0.000	0.000	0.000
	Chen (%)^d^	(1.5 ± 1.0) × 10^−1^	(3.9 ± 1.6) × 10^−1^	(1.2 ± 2.8) × 10^−2^	(1.5 ± 5.0) × 10^−3^	0.000	0.000	0.000

*Abbreviations:* SIB-IMRT = intensity-modulated radiation therapy with simultaneous integrated boost; SD-IMRT = standard-dose intensity-modulated radiation therapy; SIB-SD = the difference in predicting value between the SIB-IMRT and SD-IMRT plans; ESO_whole_ = the entire esophagus, including the tumor; ESO_infield_ = the portion of ESO_whole_ within the treatment field; ESO_infield-tumor_ = the portion of ESO_infield_, excluding the tumor; ESO_whole-tumor_ = the portion of ESO_whole_, excluding the tumor. Kwint = Kwint model; Wijsman = Wijsman model; Chen = Chen model. N/A = not available. *p* ^*1*^: ESO_whole_ vs. ESO_whole-tumor_; *p* ^*2*^ ESO_infield_ vs. ESO_infield-tumor_.

^a^Indicates Kwint model for predicting grade ≥ 2 acute esophageal toxicity.

^b^Indicates Kwint model for predicting grade ≥ 3 acute esophageal toxicity.

^c^Indicates Wijsman model for predicting grade ≥ 2 acute esophageal toxicity.

^d^Indicates Chen model for predicting late esophageal toxicity.

## Discussion

Whether different DEs influence the evaluation of the esophageal toxicity prediction for EC patients administered SIB-IMRT vs. SD-IMRT remains unknown. To address this issue, we employed four DEs to distinguish the changes on esophageal toxicity prediction between the SIB-IMRT and SD-IMRT dosing regimens using radiobiological models. We found that different DEs influence the esophageal toxicity prediction by up to 11.2% (grade ≥ 2 AET predicted with the Wijsman model). To the best of our knowledge, this study is the first to investigate the influence of different DEs on the esophageal toxicity prediction for EC patients received SIB-IMRT vs. SD-IMRT.

AET and LET characterized by dysphagia, odynophagia, stenosis and perforation are common radiation-induced adverse events^4, 15^ that significantly affect the quality-of-life and negatively impact the long-term survival of patients when received thoracic irradiation^16^. Compared to AET, LET is relatively rare^16^. In the definitive treatment of EC patients, the esophagus is more prone to develop these symptoms because part of the esophagus is inside the treatment field, leading to high dose irradiation during treatment. Three independent studies showed that 60% of the patients developed grade ≥ 2 AET, 40% of the patients developed grade ≥ 3 AET and 22% of the patients suffered from LET when experiencing SIB-IMRT treatment^2, 4, 5^. Accordingly, evaluation of the incidence of esophageal toxicity is important for clinical treatment.

Although two dosimetric studies demonstrated an improved benefit of SIB-IMRT compared with the SD-IMRT strategy^12, 17^, no further information on esophageal toxicity was provided in the two studies. Until recently, the DEs are not consistent for EC patients. Whether the portion of esophagus outside the treatment field should be included in the toxicity prediction is unclear. Caglar *et al*. suggested that the in-field esophagus was a new predictor for esophagitis in NSCLC patients^10^. However, other studies used the entire esophagus as the predictor^6, 7, 18^. Interestingly, we found that two DEs (ESO_whole_ and ESO_infield_) resulted in a similar trend of increase for esophageal toxicity prediction in the SIB-IMRT plans (Table 3), indicating that both of them are comparable for esophageal toxicity evaluation. However, we also found that the increase in esophageal toxicity using ESO_whole_ and ESO_infield_ was higher than that of the ESO_whole-tumor_ and ESO_infield-tumor_, particularly when the Wijsman model was used (Table 3). Therefore, more attention should be paid on a consensus on the DEs during the radiation therapy treatment for EC patients.

The results of our analysis are partly dependent on the choice of radiobiological models and parameters used. To strengthen the reliability of our data, we employed two esophageal toxicity predicting models from the literature to predict the likelihood toxicity of grade ≥ 2 AET. Interestingly, we observed that two independent models exhibited a similar trend of increase in the esophageal toxicity using the SIB-IMRT technique, although the absolute values were different between the two models. Considering that both of these models were generated from more than one hundred patients experiencing clinical treatment, we propose that these data on the prediction of esophageal toxicity are reliable. However, we observed that the increase using the Wijsman model was more remarkable than that from the Kwint model. This finding might partly reflect the different chemotherapy regimens used in the two independent investigations. Only low-dose cisplatin was used in the Kwint model^7^, whereas gemcitabine combined with cisplatinum or etoposide combined with cisplatinum were selectively delivered according to condition of the patients in the Wijsman model^6^. Because AET is enhanced with CCRT^19^, the chemotherapy regimen might partly influence our prediction of esophageal toxicity.

A significant diversity of predictors used for AET prediction have been reported in previous studies. Dose volume parameters, such as V_30_, V_40_, V_50_, V_60_, D_max_ and mean esophagus dose were reported to enable AET prediction^15, 18, 20–22^. Palma *et al*. performed a meta-analysis enrolling the largest population to date (1082 patients) to show that V_60_ emerged as the best predictor of grade ≥ 2 and grade ≥ 3 radiation-induced esophagitis with good calibration and discrimination^21^. The results indicated that the high dose region might principally contribute to the formation of esophageal toxicity. From this perspective, we infer that it is more reasonble to use ESO_whole_ and ESO_infield_, which include the GTV for esophageal toxicity prediction. However, further clinical validation studies are warranted to confirm our speculation. Recently, Wang *et al*. used a receiver operating characteristic (ROC) curve to analyze the predictive values of three methods of lung definitions for radiation pneumonitis (RP)^23^. The authors concluded that the definition of  lungs-GTV (lung subtracts GTV) might be the most accurate definition for predicting RP. We have completed a phase II study implementing the SIB-IMRT strategy combined with chemotherapy for EC patients^3^, and more accurate DE is expected after analyzing the clinical data using the ROC method.

In the past few years, many studies have developed many dosimetric parameters to predict the occurrence of grade ≥ 2 or grade ≥ 3 AET^20–22^; however, only several studies have developed models to predict their incidence^6, 7, 18, 24^. Furthermore, two independent studies have proposed models to predict the incidence of grade ≥ 2 or grade ≥ 3 esophagitis; unfortunately, both studies were conducted using 3-dimensional conformal radiation therapy (3DCRT) technique^11, 18, 24, 25^. Because IMRT has been reported as superior in delivering a more conformal dose and improving normal tissue sparing compared with 3DCRT^26, 27^, the models generated from 3DCRT might potentially limit the evaluation of AET for patients undergoing IMRT treatment. To date, AET predicting models derived from patients undergoing IMRT and chemotherapy treatment are scarce except for the Kwint and Wijsman models. Consistent with this information, we used these models for esophageal toxicity prediction in the study.

Although our study has demonstrated that different DEs influence the esophageal toxicity prediction for EC patients administered SIB-IMRT vs. SD-IMRT dosing strategies, there are some limitations. (1) The sample size of our study was a bit small to fully distinguish the changes in dosimetry and esophageal toxicity between the SIB-IMRT and SD-IMRT dosing strategies. Thus, a larger patient cohort is needed for further validation in the clinic. (2) We employed the esophagitis-predicting models from lung cancer patients, but the applicability and feasibility of these models should to be further validated. (3) Notably, only the Kwint model was used to predict grade ≥ 3 AET which might partially weaken the reliability of our results. However, to the best of our knowledge, the Kwint model is the only grade ≥ 3 AET-predicting model derived from IMRT and chemotherapy practices, and this model should be used it for prediction.

We must state that AET and LET is dose relavent. With standard dose, SIB-IMRT strategy for EC patients was reportly feasible without increasing AET or LET at neoadjuvant and adjuvant CCRT settings^28, 29^.

## Conclusions

In summary, our study demonstrated that different DEs influence the esophageal toxicity prediction for EC patients administered SIB-IMRT vs. SD-IMRT. Our results require further validation in clinical samples.
